# A Challenging Case of Inferior Vena Cava Compression in an Adult Polycystic Kidney Disease Patient. A Case Report

**DOI:** 10.1002/ccr3.70283

**Published:** 2025-03-02

**Authors:** Ahmad Matarneh, Abdelraouf Akkari, Sundus Sardar, Ronald Miller, Navin Verma, Nasrollah Ghahramani, Umar Farooq

**Affiliations:** ^1^ Department of Nephrology Penn State Milton S Hershey Medical Center Hershey Pennsylvania USA

**Keywords:** adult polycystic kidney disease, end stage renal disease, hemodialysis, inferior vena cava compression

## Abstract

Adult polycystic kidney disease (ADPKD) is a multi‐system genetic disorder characterized by the development and progressive enlargement of fluid‐filled cysts in both kidneys, along with other organs. As one of the main causes of kidney failure, ADPKD can progress to end‐stage renal disease (ESRD), with over 50% of affected individuals progressing to ESRD by age 50. The symptoms in ADPKD are variable, with some patients experiencing nonspecific signs, while others present with symptoms related to the mass effect of enlarged kidneys on surrounding structures. This case report highlights an unusual presentation of ADPKD in a patient who developed symptoms of inferior vena cava (IVC) compression. Remarkably, these symptoms improved after bilateral nephrectomies, suggesting that bilateral nephrectomy would provide help in these situations.

**Summary**

- Adult polycystic kidney disease (ADPKD) can present atypically, such as with symptoms of inferior vena cava (IVC) compression due to enlarged kidneys.
- This case highlights significant symptom improvement following bilateral nephrectomies, suggesting nephrectomy as a potential intervention for ADPKD patients experiencing severe IVC compression and related complications.

## 1 Introduction

Adult polycystic kidney disease (ADPKD) is an autosomal dominant inherited condition caused by mutations in the PKD1 gene. It is characterized by the formation of cysts within the kidneys, as well as other organs [1]. It may be asymptomatic initially, but as cysts enlarge, it can cause a wide range of symptoms related to the disease's mass effect. Abdominal discomfort, hematuria, and renal colic due to renal calculi are among the most common symptoms [2]. Massive kidney enlargement can lead to compression of nearby vascular structures, notably the IVC. IVC compression can result in impaired venous return, leading to venous stasis, congestion, and edema in the lower limbs [3]. Diagnosis of IVC compression typically requires imaging studies, such as abdominal CT scans with contrast, to assess the extent of kidney enlargement and vascular involvement [4]. This report highlights the clinical significance of identifying inferior vena cava (IVC) compression in patients with autosomal dominant polycystic kidney disease (ADPKD) and its associated hemodynamic and systemic effects. It presents the case of a 51‐year‐old female with ESRD due to ADPKD who experienced persistent lower limb edema with cramping during hemodialysis. Imaging revealed significant IVC compression due to the mass effect of her kidneys. She improved after bilateral nephrectomies.

## 2 Case History and Examination

The patient is a 51‐year‐old female with a history of ESRD secondary to ADPKD, for which she has been receiving maintenance hemodialysis (HD) for 4 years. Her ADPKD was diagnosed back in 1992 on the basis of a CT scan of the abdomen; she also has a strong family history of ADPKD. Her ADPKD was managed with Lisinopril and conservative measures. She presented to the HD clinic with complaints of persistent bilateral lower limb edema, which did not respond to adjustments in her estimated dry weight (EDW) and increasing ultrafiltration during dialysis sessions. Despite her chest examination being clear with no evidence of fluid overload in the lungs, her edema persisted. Attempts to modify her dialysis prescription to achieve better fluid removal were unsuccessful, and she continued to experience lower limb edema as well as cramping during HD.

On physical examination, the patient had a distended abdomen, though bowel sounds were normal. There was no tenderness on palpation, but the kidneys appeared to be markedly enlarged.

### 2.1 Differential Diagnosis and Investigations, Treatment

**FIGURE 1:**
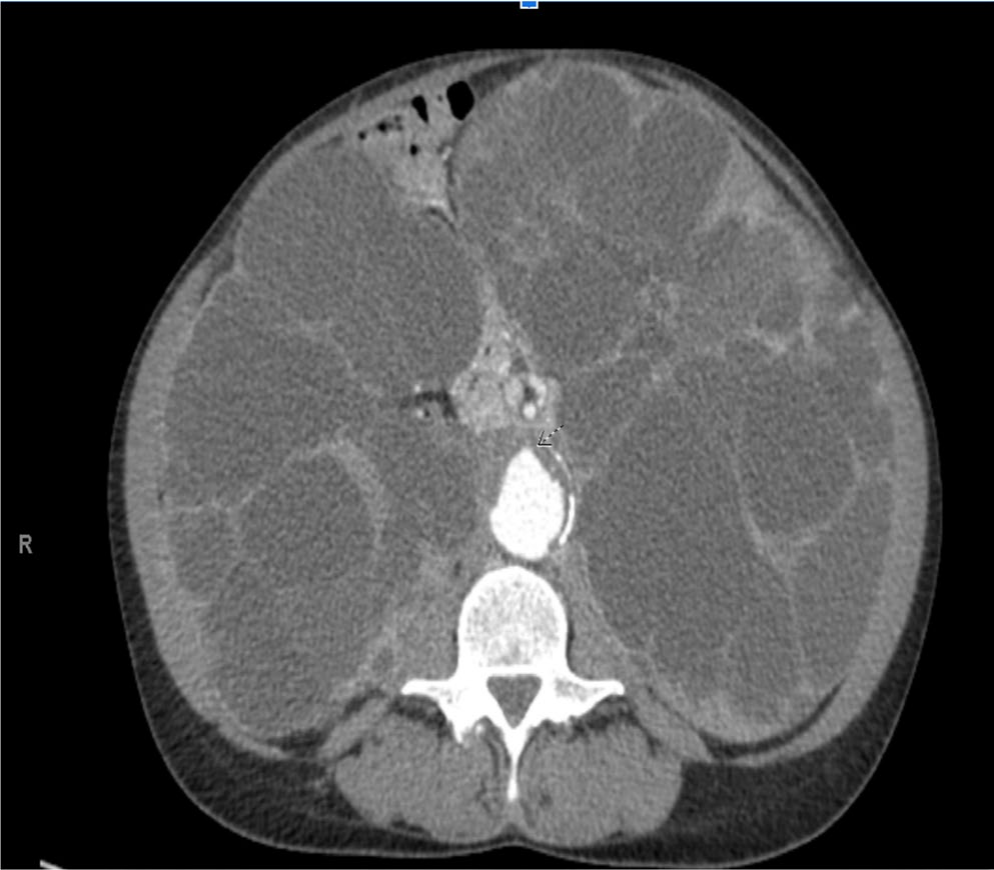
CT scan of the abdomen, axial view showing bilateral enlarged kidneys, more on the left, causing compression of theIVC.

**FIGURE 2:**
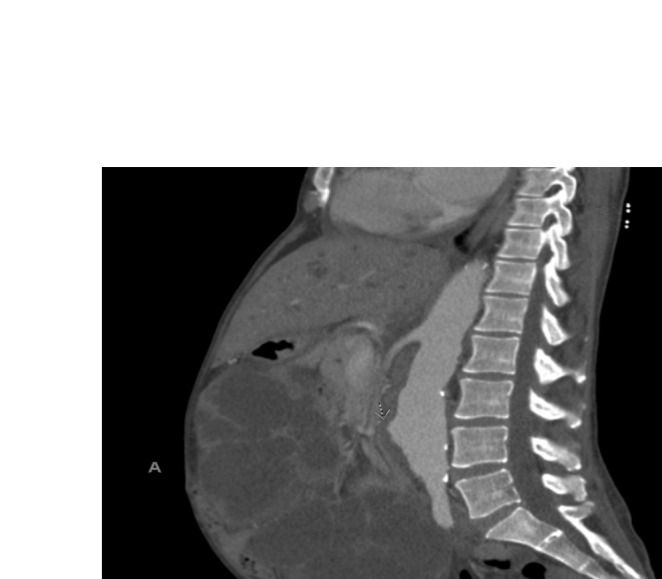
CT scan of the abdomen, sagittal view, showing IVC compression and mass effect.

**FIGURE 3:**
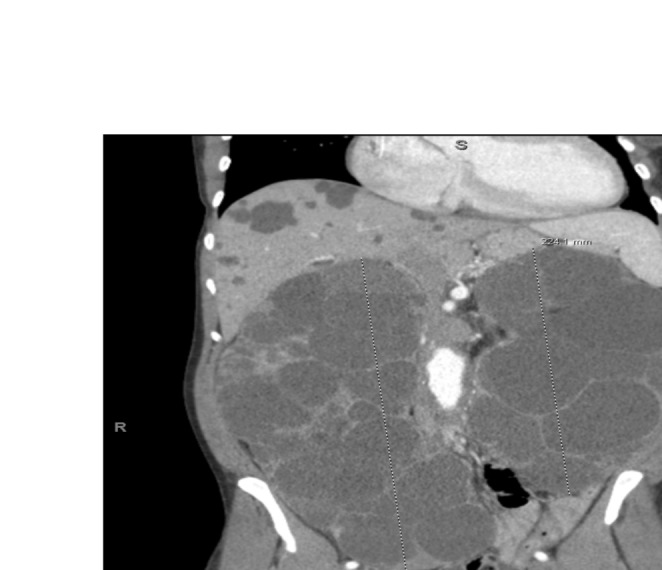
Coronal view, showing bilateral enlarged kidneys causing IVC compression.

Given her ongoing symptoms and the lack of response to fluid management changes, there was a suspicion that her symptoms could be related to IVC compression caused by enlarged kidneys. A bedside ultrasound was performed, which revealed large, cyst‐filled kidneys. Consequently, a contrast‐enhanced CT scan of the abdomen was ordered, confirming the presence of massively enlarged kidneys, with measurements of 22 × 22 × 12 cm on the left side and 29 × 19 × 12 cm on the right. The CT scan further revealed compression of the IVC, likely resulting in venous congestion and contributing to her lower limb edema and dialysis‐associated cramping. (Figures 1, 2, 3).

### 2.2 Outcome and Follow Up

**FIGURE 4:**
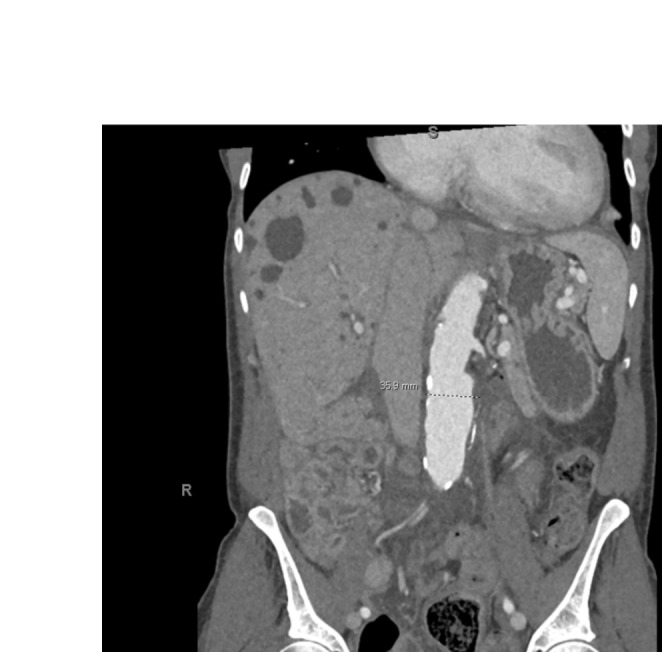
CT scan of the abdomen, coronal view showing postoperative changes and interval bilateral nephrectomy.

Following this diagnosis, the patient was referred to the urology team, who recommended bilateral nephrectomies to relieve the IVC compression. The patient subsequently underwent the surgical procedure, which was successful, and her symptoms improved significantly postoperatively. She continued to undergo HD without further episodes of cramping or persistent edema. At follow‐up, the patient reported a substantial improvement in her quality of life and an absence of her previous symptoms. A CT scan was done for follow up, which showed resolution of IVC compression and post‐nephrectomy changes (Figure 4).

## 3 Discussion

This study aimed to highlight the risks of mass effects in autosomal dominant polycystic kidney disease (ADPKD), with a particular focus on inferior vena cava (IVC) compression and its management. ADPKD is among the most common genetic kidney diseases and a leading cause of ESRD, affecting approximately 1 in 400–1 in 1000 individuals worldwide [5]. The disease is characterized by gradual kidney enlargement due to cyst growth, which often results in progressive renal failure. As cysts become more enlarged, they compress surrounding renal parenchyma, ultimately compromising renal function [6]. In advanced cases, ADPKD can lead to ESRD, necessitating renal replacement therapies such as dialysis or transplantation. In addition to renal involvement, ADPKD patients may experience complications involving the liver, pancreas, and vascular system, with a significant number developing cardiovascular issues such as hypertension and vascular aneurysms [7].

When kidney enlargement is substantial, the cystic masses can compress nearby structures, including the gastrointestinal tract and vascular structures such as the IVC and, in rare instances, the aorta [8]. IVC compression due to ADPKD‐related kidney enlargement is uncommon but clinically important, as it can mimic other conditions, including volume overload, by presenting with edema and circulatory congestion in the lower extremities. Symptoms of IVC compression may include edema, venous stasis, and discomfort due to the increased pressure in the lower extremity veins, which impairs normal venous return to the heart [9]. This can create diagnostic challenges in ESRD patients on HD, where fluid management is critical and symptoms are often attributed to volume overload or intradialytic cramping.

The case presented here describes an unusual presentation of ADPKD with IVC compression that mimicked symptoms of volume overload. Diagnostic imaging confirmed the mass effect from the enlarged kidneys on the IVC, a finding that guided the decision to pursue bilateral nephrectomies as a therapeutic intervention. Following nephrectomy, the patient's symptoms resolved, indicating that the mass effect was indeed the primary cause of her lower limb edema and HD‐related cramping. This case sheds light on the importance of considering mass effect and vascular compression as potential contributors to symptoms in ADPKD patients, particularly those with ESRD where typical volume management strategies may not alleviate symptoms.

While the exact incidence of IVC compression in ADPKD remains unclear, reports suggest that it is a rare but potentially underdiagnosed complication in patients with extensive kidney enlargement [10]. This case reinforces the role of imaging in assessing symptomatic ADPKD patients with atypical presentations and provides support for considering surgical interventions, such as nephrectomy, when vascular compression leads to significant clinical symptoms.

## 4 Conclusion

Autosomal Dominant Polycystic Kidney Disease (ADPKD) is a prevalent condition that can lead to End‐Stage Renal Disease (ESRD), presenting a range of symptoms from asymptomatic cases to those with significant mass effects. When IVC (inferior vena cava) compression occurs, it may produce nonspecific symptoms that complicate diagnosis. Therefore, timely imaging is crucial for accurate diagnosis and management. Surgical intervention leads to the alleviation of symptoms.

## Author Contributions

**Ahmad Matarneh:** conceptualization, writing – original draft, writing – review and editing. **Abdelraouf Akkari:** conceptualization, writing – original draft, writing – review and editing. **Sundus Sardar:** conceptualization, writing – original draft, writing – review and editing. **Ronald Miller:** conceptualization, writing – original draft, writing – review and editing. **Navin Verma:** conceptualization, writing – original draft, writing – review and editing. **Nasrollah Ghahramani:** conceptualization, writing – original draft, writing – review and editing. **Umar Farooq:** conceptualization, writing – original draft, writing – review and editing.

## Consent

Written informed consent was obtained from the patient to publish this report in accordance with the journal's patient consent policy.

## Conflicts of Interest

The authors declare no conflicts of interest.

## Data Availability

Data included in this manuscript will be available upon a reasonable request.
